# Continuous-Flow Separation and Efficient Concentration of Foodborne Bacteria from Large Volume Using Nickel Nanowire Bridge in Microfluidic Chip

**DOI:** 10.3390/mi10100644

**Published:** 2019-09-25

**Authors:** Xiaoting Huo, Qi Chen, Lei Wang, Gaozhe Cai, Wuzhen Qi, Zengzilu Xia, Weijia Wen, Jianhan Lin

**Affiliations:** 1 Key Laboratory of Agricultural Information Acquisition Technology, Ministry of Agriculture and Rural Affairs, China Agricultural University, Beijing 100083, China; huoxiaoting@cau.edu.cn (X.H.); wanglei123@cau.edu.cn (L.W.); 2 Key Laboratory on Modern Precision Agriculture System Integration Research, Ministry of Education, China Agricultural University, Beijing 100083, China; gaozhe@cau.edu.cn (G.C.); wuzhen.qi@cau.edu.cn (W.Q.); 3 College of Food Science and Technology, Hainan University, Haikou 570228, China; qichen@hainanu.edu.cn; 4 Key Laboratory of Biorheological Science and Technology of the Ministry of Education, College of Bioengineering, Chongqing University, Chongqing 400044, China; 5 Department of Physics, Hong Kong University of Science and Technology, Clear Water Bay, Kowloon, Hong Kong; phwen@ust.hk

**Keywords:** immunomagnetic separation, nickel nanowires bridge, large-volume sample, microfluidic chip, foodborne bacteria

## Abstract

Separation and concentration of target bacteria has become essential to sensitive and accurate detection of foodborne bacteria to ensure food safety. In this study, we developed a bacterial separation system for continuous-flow separation and efficient concentration of foodborne bacteria from large volume using a nickel nanowire (NiNW) bridge in the microfluidic chip. The synthesized NiNWs were first modified with the antibodies against the target bacteria and injected into the microfluidic channel to form the NiNW bridge in the presence of the external arc magnetic field. Then, the large volume of bacterial sample was continuous-flow injected to the channel, resulting in specific capture of the target bacteria by the antibodies on the NiNW bridge to form the NiNW–bacteria complexes. Finally, these complexes were flushed out of the channel and concentrated in a lower volume of buffer solution, after the magnetic field was removed. This bacterial separation system was able to separate up to 74% of target bacteria from 10 mL of bacterial sample at low concentrations of ≤10^2^ CFU/mL in 3 h, and has the potential to separate other pathogenic bacteria from large volumes of food samples by changing the antibodies.

## 1. Introduction

Foodborne diseases pose a great threat to human health [1,2,3]. According to the report of the World Health Organization (WHO) in 2015, food contamination was responsible for illnesses caused by foodborne pathogens [4]. Unsafe foods might cause the deaths of an estimated 2 million people annually [5]. To date, currently available methods to detect foodborne bacteria mainly include culture plating (culture) [6], polymerase chain reaction (PCR) [7,8], and enzyme-linked immune-sorbent assay (ELISA) [9,10], etc. Due to the complex background of food matrix and very low concentration of pathogenic bacteria in foods for routine screening, separation and concentration of target bacteria has often been used prior to detection of bacteria and become essential to ensure the sensitivity and accuracy of bacterial detection. However, the existing bacterial separation methods, such as filtration and centrifugation, lack specificity. More importantly, they cannot be used for in-field applications. Therefore, it is of great importance to develop new, rapid, and efficient methods to separate and concentrate target bacteria from complex food matrix.

In the past two decades, immune magnetic separation has been widely used for specific separation of target bacteria with good separation efficiency [11]. It often first uses magnetic materials modified with biological recognition elements (such as antibodies) to capture the targets, then applies a magnetic field to capture the magnetized targets while removing the sample background, and finally re-suspends the targets in a smaller volume of buffer solution to obtain the purified and concentrated targets [12]. With fast development of nanomaterial technology in recent years, magnetic nanoparticles (MNPs) have been frequently reported for immunomagnetic separation with higher separation efficiency of foodborne bacteria due to their larger specific surface, better water solubility and less steric hindrance [13]. Many studies combining immunomagnetic separation with biosensors have been reported to detect foodborne bacteria as low as 10^2^ CFU/mL [14,15]. Since very low concentration of pathogenic bacteria, at the level of 10^0^ CFU/mL, are present in real foods for routine screening and not detectable using the existing bacterial detection methods, an effective way to further improve the sensitivity of these detection methods is to separate and concentrate the target bacteria from a larger volume of food sample to obtain more bacterial cells. However, the magnetic field attenuates very quickly, resulting in narrow range for capturing the magnetic nanoparticles, thus the conventional magnetic separation method can only handle a small volume (≤1 mL) of samples. In the past decade, many efforts on new immunomagnetic separation methods were made by researchers to specifically separate target bacteria from large volume. One typical example is high gradient magnetic separation (HGMS) [16], which used an external magnetic field to magnetize soft magnetic materials (such as small iron balls, iron rods, etc.) in a separation channel, producing local high gradient magnetic fields around the magnetic materials, which could capture the immune magnetic materials for specifically reacting with the targets when they flowed through the channel. However, this HGMS method was easily subject to blocking of the channel by the large-sized particles in food matrix, greatly limiting its practical applications. Another typical example is magnetophoretic separation [17], which first employed magnetic nanoparticles to capture the target bacteria in large volume, then continuous-flow separated the magnetic bacteria from the non-magnetic sample background in the presence of a gradient magnetic field. Although magnetophoretic separation was able to handle large volume of sample, it needed a large amount of MNPs to ensure sufficient reaction between the MNPs and the target bacteria, resulting in high cost. Recently, some new immunomagnetic flow separation methods were reported to continuous-flow separate target bacteria from large volume of samples by immobilizing magnetic nanoparticles at fixed places to capture the flowing target bacteria. An interesting study was reported by Lee, et al. [18], which developed a 3D-printed spiral channel to capture the MNPs for continuous-flow separation of pathogenic bacteria from large volume (10 mL) of food samples. Combined with adenosine triphosphate (ATP) detection, this separation method could detect the target *Salmonella* cells as low as 10 CFU/mL in 3 min. Moreover, the forming of magnetic particle chains in separation channel was demonstrated to enhance separation efficiency of target bacteria [19]. In addition, magnetic nickel nanowires (NiNWs) with high aspect ratio and shape anisotropic properties, which could be synthesized using chemical vapor deposition [20,21], electrochemical deposition [22,23], electrospinning [24,25], microwave-assisted process [26,27] and solvothermal methods [28,29], were reported for manipulation and separation of magnetic cells without the use of strong magnetic field. Therefore, the combination of magnetic flow separation and the magnetic NiNWs might be promising to develop efficient methods for continuous-flow separation of target bacteria from large volume of sample.

**Figure 1:**
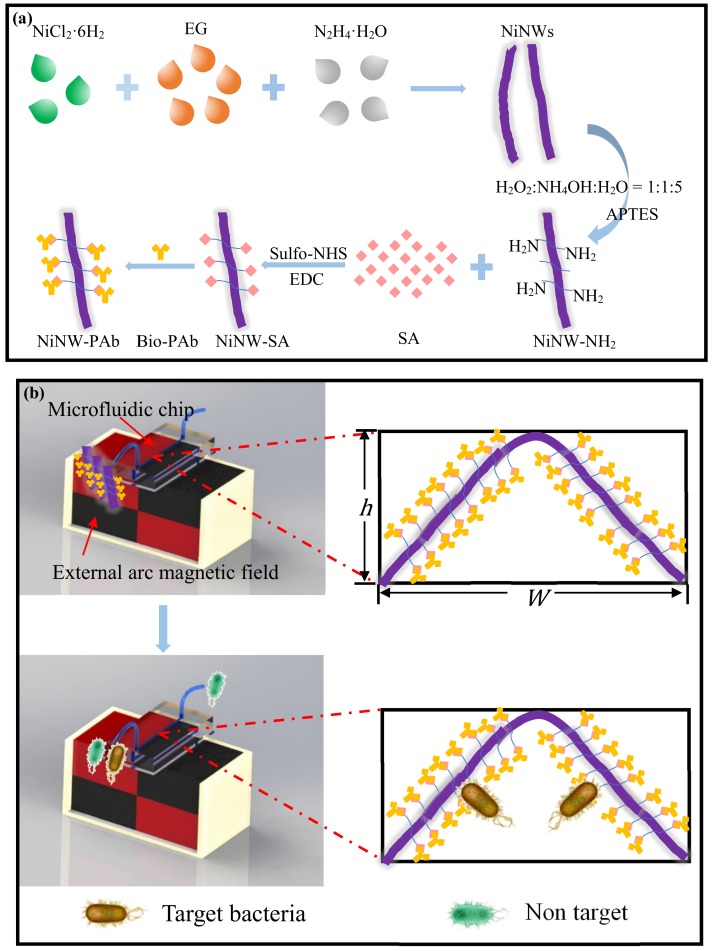
(**a**) Schematic of the synthesis of immune nickel nanowires (NiNWs); (**b**) Schematic of continuous-flow separation of the target bacteria using the NiNW bridge in the microfluidic chip.

In this study, we developed a bacterial separation system for continuous-flow separation and efficient concentration of target bacteria from large volume of sample using immune nickel nanowires as capture bridge in microfluidic chip. As shown in Figure 1, the NiNWs were first synthesized using the one-step synthesis method and immobilized with the antibodies against target bacteria through ethylcarbodiimide hydrochloride (EDC)/ N-hydroxy-succinimide (NHS) method. Then, the immune NiNWs were injected into the microfluidic channel in the presence of the external arc magnetic field to form the NiNW bridge. Finally, large volume of bacterial sample was continuous-flow injected to the channel, resulting in the specific capture of the target bacteria by the antibodies on the NiNW bridge through the antigen-antibody binding. After the magnetic field was removed, the target bacteria were flushed out of the channel with a smaller volume of phosphate-buffered saline (PBS) solution to obtain the purified and concentrated bacterial sample.

## 2. Materials and Methods

### 2.1. Materials

Nickel (II) chloride hexahydrate (NiCl_2_·6H_2_O, 99.9%), ethylene glycol (EG, 99.8%), hydrazine monohydrate (N_2_H_4_·H_2_O, 98%), and poly (vinylpyrrolidone) (PVP, MW 40,000) were obtained from Sigma Aldrich (St. Louis, MO, USA) to synthesize the nickel nanowires. Amino Propyl Triethoxy Silane (APTES), hydrogen peroxide (H_2_O_2_), and ammonium hydroxide (NH_4_OH) were purchased from Sinopharm Chemical (Shanghai, China) to functionalize the NiNWs with amino groups. 1-(3-Dimethylaminopropyl)-3-ethylcarbodiimide hydrochloride (EDC·HCl) and N-hydroxy-succinimide sodium salt (sulfo-NHS) from Sigma Aldrich and streptavidin from Hualan Chemical (Shanghai, China) were used to modify the NiNWs with streptavidin. Biotin-fluorescein isothiocyanate from Sigma Aldrich was used to observe the modification of streptavidin on the NiNWs. Bovine serum albumin (BSA) from Sigma Aldrich was used to block the non-specific binding sites. Deionized water (18.2 MΩ·cm) was produced by Advantage A10 (Millipore, Billerica, MA, USA). The concentrated phosphate-buffered saline (PBS, P5493) from Sigma Aldrich was 10 times diluted with deionized water to prepare the PBS solution (pH 7.4, 0.01 M). The silicone elastomer kit (Sylgard 184, Dow Corning, Auburn, MI, USA) was used to fabricate the poly (dimethoxy) silane (PDMS) channel. The 3D printer (Objet24, Stratasys, Eden Prairie, MN, USA) was used to fabricate the mold of the channel.

### 2.2. Fabrication of the Microfluidic Chip

The microfluidic chip is a key component of this proposed bacterial separation system. The chip mainly included a straight separation channel with the length of 55 mm, the width of 700 µm, and the height of 200 µm and was used with an arc magnetic field to capture the immune NiNWs in the microfluidic channel to form the NiNW bridge for continuous-flow separation of the target bacteria while they flowed through the channel.

The microfluidic chip was fabricated based on 3D printing and surface plasma bonding. First, the mold of the microfluidic channel was designed by SolidWorks and fabricated using the 3D printer, followed by immersing in 5% NaOH for 30 min to thoroughly remove the surplus supporting material. Then, the PDMS prepolymer and the curing agent were mixed at a ratio of 10:1 and cast into the mold after degassing in vacuum for 20 min, followed by curing at 65 °C overnight. Finally, the PDMS channel was peeled from the mold and bonded with a clean glass pretreated using oxygen plasma (Harrick Plasma, Ithaca, NY, USA) to fabricate the microfluidic chip. The whole microfluidic chip was 65 mm long, 6 mm wide, and 40 mm thick.

### 2.3. Synthesis of the Nickel Nanowires

The nickel nanowires are the key material for the bacterial separation system to specifically separate the target bacteria, which were synthesized according to our previous studies [30,31] with slight modifications. First, 75 μL of 1 M aqueous NiCl_2_·6H_2_O (99.9%) and 15 mL of EG (99.8%) were mixed and heated to 100 °C, followed by adding 0.5 mL of N_2_H_4_·H_2_O drop by drop. The temperature was remained at 100 °C for ~30 min until the dark gray product was formed and floated on the solution’s surface. The product was then washed with deionized water and anhydrous ethanol three times through magnetic decantation, and finally dispersed in ethanol. PVP (0.03%, w/v) in EG solution was used as the solvent in this study instead of pure EG due to its strong control on the size and the stability of the NiNWs.

### 2.4. Functionalization of the Nickel Nanowires

The separation of the target bacteria is based on the immune reaction between the target bacteria in the sample and the antibodies on the nickel nanowires. First, 500 μL of the NiNWs (1 mg/mL) were immersed in 700 μL of the mixture of H_2_O_2_/NH_4_OH/H_2_O (1:1:5, v/v) for 30 min at 80 °C, followed by rinsing with deionized water and drying at 65 °C for 15 min to functionalize the NiNWs with the -OH groups on their surface. Then, the NiNWs were silanized by incubating with 1 mL of 1% APTES in ethanol overnight to functionalize the NiNWs with the -NH_2_ groups. After 189 μL of 0.04 M EDC and 750 μL of 0.01 M sulfo-NHS were added with 50 μg of streptavidin and incubated for 3 h with gentle stirring, the streptavidin-coated NiNWs were washed with PBS twice, blocked by 1% BSA for 1 h, and finally dispersed in 500 μL of PBS.

For the modification of the streptavidin-coated NiNWs with the antibodies, 100 μL of the prepared NiNWs were first washed with PBS twice and re-suspended in 500 μL of 10 mM sterile PBS. Then, 4 μg of the biotinylated antibodies against the target bacteria were added and incubated at 15 rpm for 30 min, resulting in the modification of the antibodies onto the NiNWs through streptavidin-biotin binding. After magnetic separation to remove the excessive antibodies, the immune NiNWs were finally re-suspended in 500 μL of sterile PBS, and stored at 4 °C for further use.

### 2.5. Culture and Enumeration of the Target Bacteria

*E. coli* O157:H7 (ATCC 43888) and *Salmonella* Typhimurium (ATCC 14028) were used as research models to evaluate the proposed bacterial separation system. They were first incubated in the *Luria-Bertani* (LB) medium (Aoboxing Biotech, Beijing, China) for 13–16 h at 37 °C with shaking at 180 rpm. Then, the bacterial cultures were serially diluted with PBS to obtain the bacterial samples at the concentrations from 10^0^ to 10^4^ CFU/mL, respectively.

For enumeration of the bacterial samples, 100 μL of each dilution of the samples was surface plated onto one LB agar plate, and then incubated at 37 °C for 16 h before the colonies were counted. The concentration of the bacteria was determined by counting the viable colonies on the plate.

### 2.6. Separation and Concentration of the Target Bacteria

The separation and concentration of the target bacteria is based on specific binding between the antibodies on the NiNW bridge and the bacteria flowing through the microfluidic chip. To verify the superiority of this proposed bacterial separation system, the conventional magnetic separation in centrifuge tube using the same amount of the NiNWs and the MNPs was conducted in parallel, respectively. For the conventional bacterial separation, 900 μL of the bacterial sample at each concentration of 10^1^–10^4^ CFU/mL was first mixed with 100 μL of the immune NiNWs (1 mg/mL) or the immune MNPs (1 mg/mL) for 45 min at 15 rpm in the sterile centrifuge tube, which was blocked with 1% BSA for 30 min and washed with PBS prior to use for minimizing the non-specific adsorption, resulting in the formation of the NiNW–bacteria or MNP–bacteria complexes. These complexes were then magnetically separated to remove the sample background, and finally culture plated to determine separation efficiency of the target bacteria, which was defined as the ratio of the number of the separated bacteria to that of the original bacteria.

For continuous-flow separation of the target bacteria from large volume of sample, 10 mL of the bacterial samples with different concentrations of 10^0^–10^4^ CFU/mL were prepared and the microfluidic chip was blocked by 1% BSA for 30 min and washed with PBS prior to test. First, 500 μL of the immune NiNWs were continuous-flow injected into the microfluidic chip to form the NiNW bridge in the presence of the arc magnetic field at the flow rate of 200 µL/min using the syringe pump (Pump 11 Elite, Harvard Apparatus, Holliston, MA, USA). Then, 10 mL of the bacterial sample with each concentration of 10^0^–10^4^ CFU/mL was continuous-flow injected into the channel at the flow rate of 60 µL/min (see Figure S1), allowing the antibodies on the NiNW bridge to capture the target bacteria and thus form the NiNW–bacteria complexes. After washing with PBS, the magnetic field was removed and the NiNW–bacteria complexes were flushed out of the channel with 1 mL of PBS at the flow rate of 400 μL/min, followed by culture plating to determine the number of the target bacteria.

## 3. Results

### 3.1. Simulation of the Magnetic Field

The magnetic field plays an important role in the distribution of the magnetic NiNWs in the microfluidic channel, and thus is a key to improve separation efficiency of the target bacteria. In the reported studies [32] on continuous-flow immunomagnetic separation of target bacteria, the immune MNPs were often captured by the external magnetic field and aggregated against one side of the microfluidic channel to separate the target bacteria in the flowing sample. However, the flowing sample was generally in laminar regime, resulting in the possible capture of only a small part of the targets that flowed closely to the fixed MNPs. Due to the fact that the height (~100 μm or more) of the microfluidic channel was often much larger than that (10 μm or less) of the MNPs, the separation efficiency of the target bacteria was always very low (~10% or less). Thus, the key to improve the separation efficiency was to increase the capture range of the MNPs. One effective way was to use much more MNPs; however, this would significantly increase the cost and result in impractical applications. Thus, an alternative way was proposed in this study by developing an appropriate magnetic field to make the limited amount of magnetic materials distribute more uniformly in the cross section of the channel to increase the opportunity for the antibodies on the NiNWs to react with the flowing target bacteria.

**Figure 2:**
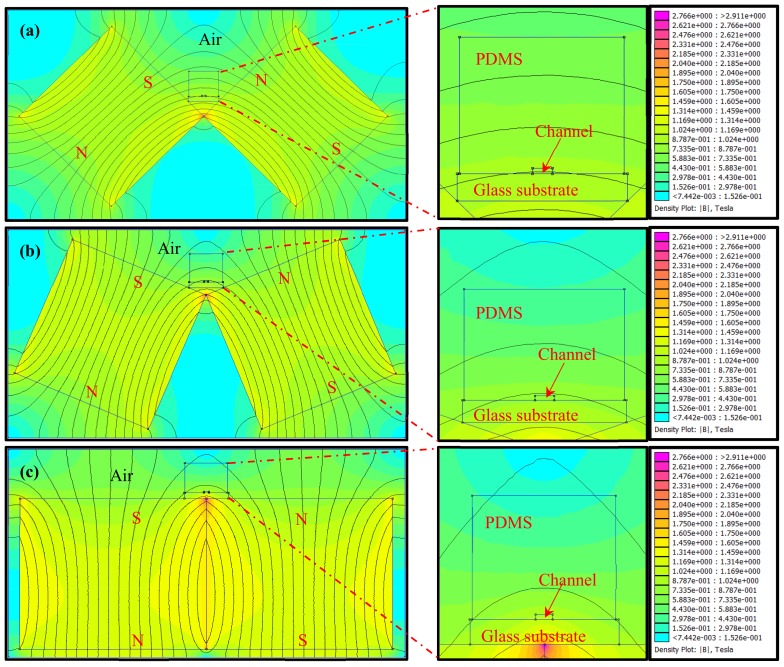
The simulation of the arc magnetic fields with different angles of (**a**) 90° (**b**) 135° and (**c**) 180°.

To develop a suitable magnetic field for controlling the formation of the NiNW bridge to occupy more space in the microfluidic channel, the Finite Element Method Magnetics (FEMM) software was used for simulation of the magnetic field. Since the NiNWs have the tendency to distribute along the magnetic field lines, an easy and effective way is to set up an arc magnetic field using two attractive magnets to form the NiNW bridge in the microfluidic channel. Figure 2 showed three setups of the magnetic field with different angles between one magnet and the other: 90° (Figure 2a), 135° (Figure 2b), and 180° (Figure 2c). It is obviously seen that the magnetic fields in the channel are arc-shaped with different radians. When the angle between the two magnets changes from 90° to 180°, the curvature of the arc magnetic field lines in the channel increases from 0.19 to 2.63, and the curvature radium decreases from 5.37 mm to 0.38 mm. To further compare these three setups, the height (*h*) of the magnetic field line passing through the left and right bottoms of the channel at the center of the channel (see Figure 1) was used to evaluate the space occupation of the NiNWs in the channel, and could be expressed as

(1) $$h=r-\sqrt{r^{2}-{\frac{w}{4}}^{2}}$$

where ***w*** is the width of the channel, ***r*** is the curvature radium of the magnetic field line. The heights for the angles at 90°, 135° and 180° were calculated to be ~12, ~50 and ~235 μm respectively, indicating that the angle of 180° had the maximum space occupation. Thus, the setup of the magnetic field with the angle of 180° was used in this study. Moreover, the image of the NiNW bridge was taken to verify its formation and shown in Figure S2.

### 3.2. Characterization of the Nickel Nanowires

**Figure 3:**
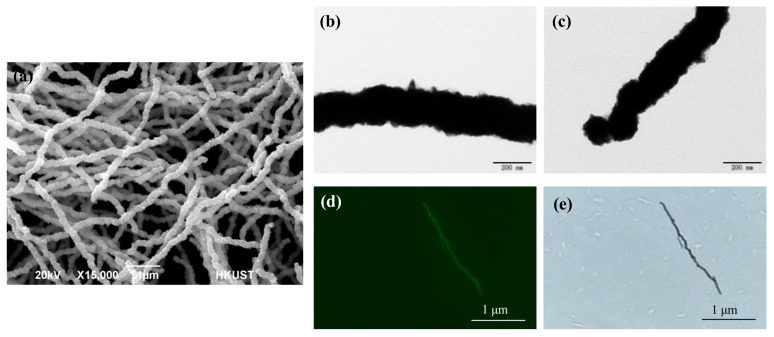
(**a**) The scanning electron microscopy (SEM) image of the NiNWs; (**b–c**) The TEM images of the NiNW; (**d**) The microscopic image of a fluorescent NiNW at fluorescent mode; (**e**) The microscopic image of the streptavidin-coated NiNW at bright mode.

The nickel nanowires are the most important material to the bacterial separation system. Both transmission electron microscopy (TEM) and scanning electron microscopy (SEM) were used to characterize the NiNWs. As shown in Figure 3a, the averaged length and diameter of the NiNWs based on the calculation of 50 NiNWs are 14.80 ± 3.65 μm and 190 ± 28 nm, respectively. The aspect ratio of the NiNWs is 78 ± 22. It is also seen that there are some small spiked structures on the surface of the NiNWs (Figure 3b) and some nanoparticles at the end of the NiNWs (Figure 3c), indicating that the NiNWs have a high heterogeneous surface and a large shape anisotropy, which can increase specific surface and thus be modified with more antibodies.

The loading capacity of the antibodies onto the NiNWs is crucial to the separation of the target bacteria. Thus, after the NiNWs were functionalized with streptavidin, they were first conjugated with the biotinylated fluorescein and then observed using the Eclipse TE300 inverted epifluorescence microscope (Nikon, Tokyo, Japan) to check the loading capacity of the NiNWs. The images of a streptavidin-coated NiNW and a fluorescent NiNW are shown in Figure 3d–e. It is seen that the whole NiNW is conjugated with the fluorescein, indicating full loading of streptavidin on the NiNW, i.e., the biotinylated antibodies against the target bacteria can be modified onto the whole surface of the NiNWs through the streptavidin-biotin binding.

### 3.3. Comparison of the NiNWs and MNPs for Conventional Magnetic Separation of Bacteria

**Figure 4:**
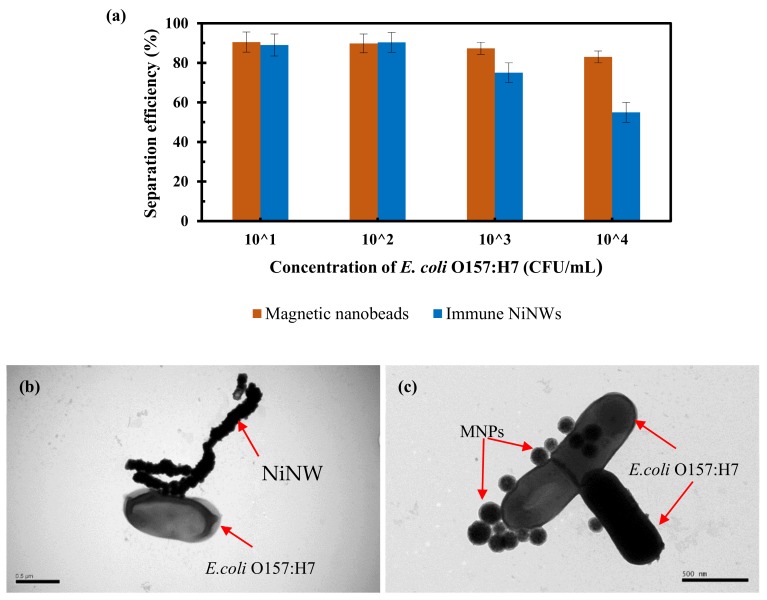
(**a**) The separation efficiency of *E. coli* O157:H7 at the concentrations of 1.2 × 10^1^–1.2 × 10^4^ CFU/mL using both the NiNWs and the MNPs; (**b**) The TEM image of the NiNW–bacteria complex; (**c**) The TEM image of the MNPs-bacteria complex.

Magnetic nanoparticles are the most often used for immunomagnetic separation of bacteria. Thus, the NiNWs were compared with the commonly used magnetic nanoparticles to separate the target bacteria using *E. coli* O157:H7 as research model. The pure bacterial cultures with the concentrations ranging from 1.2 × 10^1^ CFU/mL to 1.2 × 10^4^ CFU/mL were prepared and 1 mL of each culture for immunomagnetic separation using both the immune NiNWs and the immune MNPs in centrifuge tube. Both the original and separated number of the *E. coli* O157:H7 cells were determined using gold standard culture plating. As shown in Figure 4a, the separation efficiency (~90.2%) of the target bacteria for the NiNWs is comparable with that (~89.7%) of the MNPs at lower bacterial concentrations (1.2 × 10^1^ CFU/mL and 1.2 × 10^2^ CFU/mL), and gradually less than that of the MNPs at higher concentrations (1.2 × 10^3^ CFU/mL and 1.2 × 10^4^ CFU/mL). This indicated that the NiNWs did not have any superiority to the MNPs in the conventional magnetic separation strategy in centrifuge tube. This could be explained as follows. At the lower bacterial concentrations, both the NiNWs and the MNPs were far excessive for the capture of most target bacteria. However, at the higher concentrations, the larger NiNWs were subject to precipitate compared to the smaller MNPs, resulting in less opportunities for the antibodies on the NiNWs to react with the target bacteria in the solution. Thus, the MNPs was more suitable than the NiNWs for magnetic separation of target bacteria in centrifuge tube. However, the larger NiNWs might be possibly easier to form the bridge in the presence of the arc magnetic field according to the reported studies on the formation of the MNP chains [33], resulting in better capture of the flowing target bacteria. Moreover, to ensure successful capture of the *E. coli* O157:H7 cells using the immune NiNWs and MNPs, transmission electron microscope was performed. As shown in Figure 4b–c, the immune NiNWs and MNPs are successfully conjugated with the *E. coli* O157:H7 cells, respectively.

### 3.4. Separation of the Pure Bacteria from Large Volume in Microfluidic Chip

**Figure 5:**
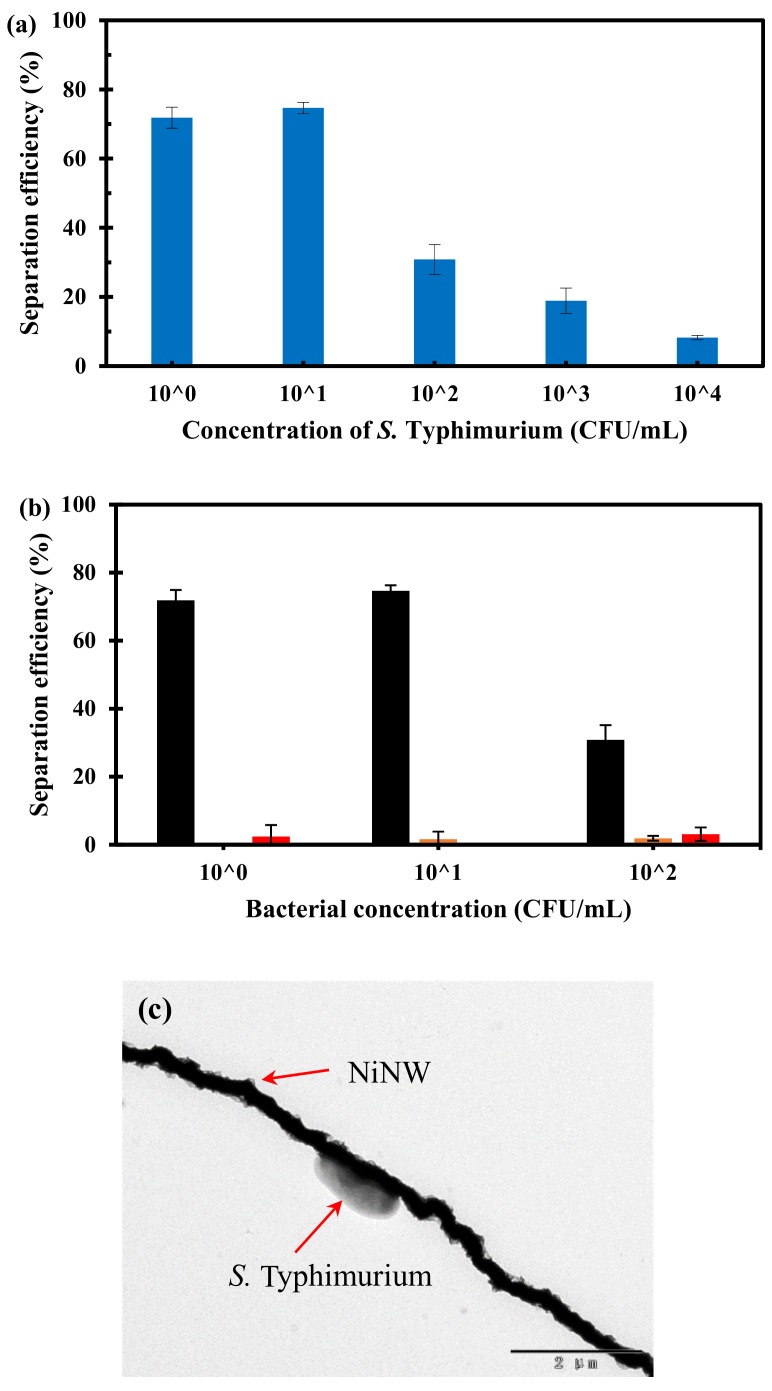
(**a**) The separation efficiency of *S.* Typhimurium at the concentrations of 1.4 × 10^0^–1.4 × 10^4^ CFU/mL using this proposed separation system; (**b**) The results for non-specific binding of *E. coli* O157:H7 and *S.* Typhimurium onto the NiNWs without antibody modification, Black: NiNWs with antibody modification for *Salmonella* separation, Orange: NiNWs without antibody modification for *Salmonella* separation, Red: NiNWs without antibody modification for *E. coli* separation; (**c**) The TEM image of the NiNW-*Salmonella* complex.

To verify the superiority of the NiNWs in the formation of the bacterial capture bridge and the versatility of the bacterial separation method, another common foodborne pathogenic bacterium, *Salmonella* Typhimurium, was used as research model. Three parallel tests on a larger volume (10 mL) of the pure *Salmonella* cells with different concentrations ranging from 1.4 × 10^0^ to 1.4 × 10^4^ CFU/mL were conducted using this proposed bacterial separation system. As shown in Figure 5a, at low concentrations (1.4 × 10^0^ CFU/mL–1.4 × 10^1^ CFU/mL), the separation efficiency of the target bacteria using the NiNWs is ~74%, while the separation efficiency decreases obviously at high concentrations (1.4 × 10^2^–1.4 × 10^4^ CFU/mL). This phenomenon could be explained as follows. When the concentration of target bacteria was low, most of the target bacteria could be captured onto the immune NiNW bridge because the binding sites were abundant, resulting in higher separation efficiency. When the concentration of the target bacteria increased, the limited binding sites might be not enough to capture all the flowing target bacteria, resulting in lower separation efficiency. Moreover, this trend was consistent with the results of the conventional magnetic separation using the NiNWs in the centrifuge tube in Figure 4a. Moreover, the non-specific binding to the NiNWs was also tested using the NiNWs without antibody modification to capture the target *S.* Typhimurium cells (8.8 × 10^0^–8.8 × 10^2^ CFU/mL) and the non-target *E. coli* O157:H7 cells (4.0 × 10^0^–4.0 × 10^2^ CFU/mL). The experimental results are shown in Figure 5b that the separation efficiencies for the NiNWs without antibodies modification are much lower than those of the immune NiNWs. According to China’s food safety national standards, most pathogenic bacteria, such as *Salmonella* Typhimurium and *E. coli* O157:H7, are not allowed to be detectable in many ready-to-eat foods, thus the bacterial concentration is generally very low in routine screening of foodborne bacteria, i.e., at the level of 10^0^ CFU/mL. Therefore, this proposed bacterial separation system was practical for specific separation of target bacteria. Moreover, to ensure successful capture of the *S.* Typhimurium cells using the NiNW bridge, transmission electron microscope was performed. As shown in Figure 5c, the immune NiNW is successfully conjugated with *S.* Typhimurium through antigen-antibody reaction.

### 3.5. Separation of the Target Bacteria in the Spiked Chicken Samples

**Figure 6:**
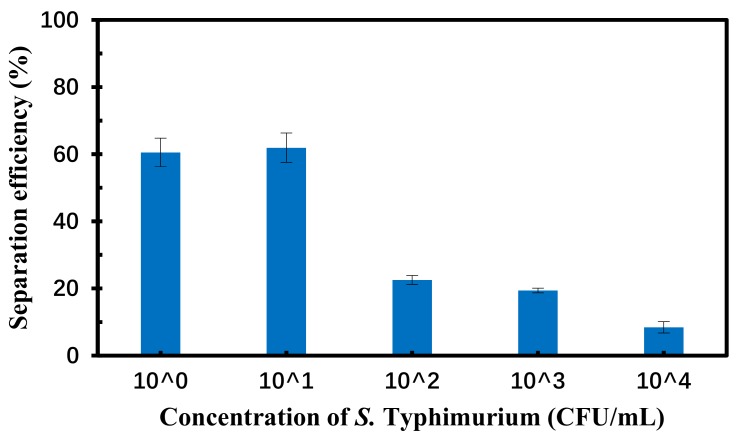
The separation efficiency of *S.* Typhimurium at the concentrations of 1.2 × 10^0^–1.2 × 10^4^ CFU/mL in the spiked chicken samples using this proposed separation system.

To further evaluate this bacterial separation system, three parallel tests on different concentrations of *Salmonella* Typhimurium in the spiked chicken carcass were conducted using this proposed system to separate the target bacteria from large volume of sample and the culture plating to determine the amount of the separated bacteria. As shown in Figure 6, the separation efficiency of *S.* Typhimurium at each concentration from 1.2 × 10^0^ to 1.2 × 10^4^ CFU/mL in the spiked chicken carcass is a little lower than that of pure cultures at the same concentration. This might be due to the complex background of the chicken carcass samples, such as proteins, lipids, and tissues, which might have impact on specific reaction between the target bacteria and the antibodies on the NiNW bridge. The trend of the separation efficiency of the target bacteria in the microfluidic channel was consistent with in the centrifugal tube, indicating that this proposed bacterial separation system was suitable for separation of target bacteria from large volume of sample.

## 4. Conclusions

In this study, we developed a bacterial separation system for continuous-flow separation and efficient concentration of foodborne bacteria from large volume using the nickel nanowires bridge in the microfluidic chip. The NiNW bridge were successfully formed in the microfluidic chip using the arc magnetic field and demonstrated to be able to continuous-flow separate ~74% of the target bacteria from 10 mL of bacterial sample at low concentrations to obtain more target bacteria than conventional magnetic separation. The separation efficiency of this proposed bacterial separation system could be further improved using more NiNWs in a longer microfluidic channel. This separation system is a promising alternative to use with the rapid detection technologies such as biosensors and PCR for sensitive routine screening of foodborne pathogens to ensure food safety. It could also be applied to separate a wide variety of biological targets by changing the biological recognition elements.

## Supplementary Materials

The following are available online at https://www.mdpi.com/2072-666X/10/10/644/s1, Figure S1: The photographs of continuous-flow separation system (a) and the microfluidic chip (b); Figure S2: The image of the NiNWs bridge.
