# Supplementary material for: Continuous-Flow Separation and Efficient Concentration of Foodborne Bacteria from Large Volume Using Nickel Nanowire Bridge in Microfluidic Chip
Source: Micromachines (Basel). 2019 Sep 25;10(10):644. doi: 10.3390/mi10100644 (PMC6843788; doi:10.3390/mi10100644)
Supplement: Supplementary file 1 [file micromachines-10-00644-s001.pdf]

Supplementary Materials: Continuous-flow separation and efficient concentration of foodborne bacteria from large volume using nickel nanowire bridge in microfluidic chip

Xiaoting Huo, Qi Chen, Lei Wang, Gaozhe Cai, Wuzhen Qi, Zengzilu Xia, Weijia Wen and Jianhan Lin

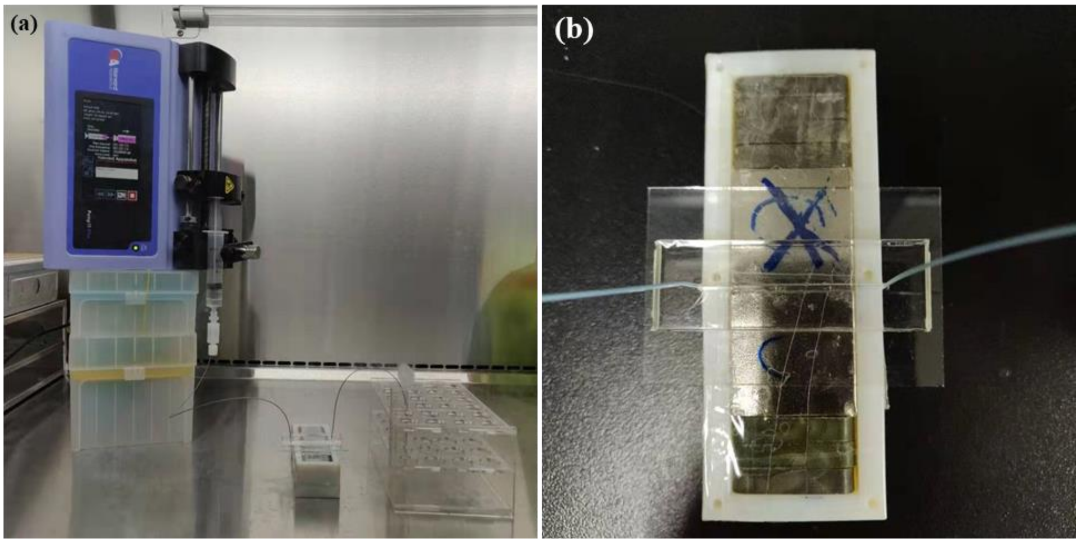

Figure S1. The photographs of continuous-flow separation system (**a**) and the microfluidic chip (**b**).

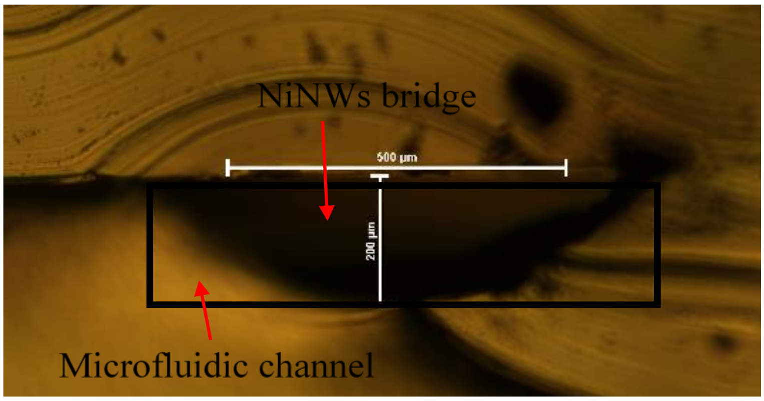

Figure S2. The image of the NiNWs bridge.
